# Effects of Regional Weather and Agricultural Practices on the Nest Survival of Northern Lapwings in France

**DOI:** 10.1002/ece3.73462

**Published:** 2026-04-17

**Authors:** Reinier F. Van den Berg, Brett K. Sandercock, Céline Le Bohec, Anna P. Nesterova

**Affiliations:** ^1^ Université de Strasbourg, CNRS, IPHC UMR7178 Strasbourg France; ^2^ Norwegian Institute for Nature Research (NINA) Trondheim Norway; ^3^ CEFE, Université de Montpellier, CNRS, EPHE, IRD Montpellier France; ^4^ Département de Biologie Polaire Centre Scientifique de Monaco Monaco Principality of Monaco; ^5^ Oréade‐Brèche Schirmeck France

**Keywords:** agriculture, camera trap, conservation, hatching success, waders

## Abstract

Northern Lapwings ( *Vanellus vanellus* ) have been in a decades‐long population decline in Europe. Previous studies have shown that declines in lapwings and other meadow‐breeding waders can be explained by reduced reproductive success instead of low adult survival. Here, we assessed pressing threats to the successful nesting of lapwings in the southern part of their breeding range, in France, which hosts an estimated 12,000–18,000 breeding pairs. We monitored nests of lapwings with nest cameras and weekly visits during the 2021 and 2022 breeding seasons at inland sites in Alsace in eastern France, and at coastal sites in Hauts‐de‐France along the English Channel in northern France. We compared nest survival rates between regions and monitoring methods, and examined the effects of the microclimate at the nest on daily nest survival. Nest survival was high overall, and at least 66% of monitored nests successfully hatched and produced nestlings. We found that nest survival rates were lower in Alsace than in Hauts‐de‐France, especially when wind speeds at the nest sites were lower. Nests equipped with pole‐mounted cameras had higher rates of nest survival, particularly in Alsace. Model‐based estimates of nest survival for a 27‐day exposure period were 0.66 and 0.59 for nests with and without camera‐monitoring at Hauts‐de‐France, but 0.43 and 0.007 for nests with and without camera‐monitoring at Alsace. The main causes of failure were damage by agricultural equipment (11% of nests), abandonment (8.2%), and depredation (7.5%). Losses to agriculture were more common in Alsace than in Hauts‐de‐France. Where nest predator species could be determined from camera images, mammals were responsible for all but one depredation event. Our study suggests that the reproductive success of lapwings in France could be increased by limiting losses to agriculture through the use of nest marking or by deploying fences to reduce mammalian predation.

## Graphical abstract

This study monitored nests of lapwings with cameras and weekly visits at inland sites in eastern France, and at coastal sites in northern France. About a third of nests failed; the main causes of failure were agricultural equipment (11%), abandonment (8.2%), and depredation (7.5%). Estimates of nest survival for a 27‐day incubation were 66% and 59% for nests with and without camera‐monitoring in northern France, but 43% and 0.7% for nests in eastern France. Lapwings breeding on agricultural fields may show high rates of nest survival, but can be hindered by adverse weather and human activity on these fields.

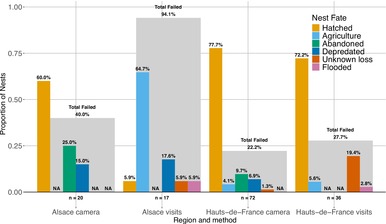

## 1 Introduction

Waders are an ecologically diverse group of birds in the order *Charadriiformes*, with declining population trends identified for about 59% of species (Koleček et al. 2021). Waders' adult survival rates usually appear to be relatively stable over time, whereas reproductive rates have not been sufficient to support stable populations (Roodbergen et al. 2012; Souchay and Schaub 2016; Franks et al. 2017; Plard et al. 2020; Ewing et al. 2023). During the breeding season, waders can be found in a diverse range of habitats, including farmland (Jóhannesdóttir et al. 2018), which exposes them to anthropogenic factors during nesting, particularly when agricultural practices include tilling or planting of crops in the fields (Kragten and De Snoo 2007; Sheldon et al. 2007). The most prominent causes of low reproductive rates have been identified as habitat loss (Leyrer et al. 2018), agricultural intensification (Donald et al. 2006), and depredation of eggs and chicks (MacDonald and Bolton 2008b; Eglington et al. 2009).

Abiotic factors, such as temperature, may also affect reproductive rates by increasing the cost of maintaining body condition (Van De Ven et al. 2019). If environmental conditions exceed a critical threshold, nests may be abandoned by the attending parents (Sharpe et al. 2019). Behavioural compensation, such as shading eggs or using water to cool down, can mitigate some of the impact of abiotic conditions (Brown and Downs 2003; Ryeland et al. 2021). Nevertheless, we can expect birds' use of breeding sites to match an optimal environmental niche (Hirzel and Le Lay 2008; Boyle et al. 2020), outside of which breeding success is expected to be limited. Rates of reproductive failure and their causes can vary significantly among breeding sites (Seymour et al. 2003; Teunissen et al. 2008), and so it is critical to assess local drivers of reproductive failure before effective conservation strategies can be developed.

Among waders, the Northern Lapwing ( *Vanellus vanellus* , hereafter ‘lapwing’) is a ground‐nesting Palearctic species breeding in open habitats. Populations are declining across much of the breeding range (Deceuninck 2001; Taylor and Grant 2004; Wilson et al. 2005; Lislevand et al. 2021; Joyeux et al. 2022). Ground‐based mammalian predators ranging in size from rats to foxes, to jackals and badgers may depredate lapwing nests (Bolton, Tyler, et al. 2007; Teunissen et al. 2008; Rickenbach et al. 2011; Männil and Ranc 2022). Avian predators such as gulls, corvids, and raptors may threaten lapwing offspring and elicit shared defensive behaviours from adults in their loose breeding colonies (Elliot 1985, Bolton, Tyler, et al. 2007; Teunissen et al. 2008; Królikowska et al. 2016).

In France, the size of the breeding population of lapwings was reported to be 12,000–18,000 pairs in 2011 (Caupenne and Trolliet 2015 in Issa and Muller 2015), down two‐thirds from 31,450–45,240 pairs in 1964 (Spitz 1964 in Joyeux et al. 2022). At a regional level, some subpopulations decreased by as much as 90% between the 1960s and the 1980s (Dubois et al. 1991). Crop fields were frequently used as nesting sites in Northern France but showed low rates of fledging (Triplet et al. 1997). However, more detailed information on reproductive success is scarce.

Since the 1960's, long‐term changes in predator numbers and habitats have affected breeding conditions for lapwings in France. Populations of red fox ( *Vulpes vulpes* ) have increased between 2003 and 2013 (Ruette et al. 2015). Fallow fields and grasslands covered about 17 million hectares in 1970, but ca. 3.8 million hectares (22.4%) were lost between 1970 and 2010 due to land‐use changes (Agreste 2012). In addition, shifts in crop types and sowing dates may have increased conflicts between breeding lapwings and agricultural practices (Schott et al. 2010; Caubel et al. 2018; Santangeli et al. 2018).

In our field study, we conducted an investigation of lapwing nesting success in two regions of France that represent endpoints along a national coastal‐continental climate gradient. Lapwing populations have declined in the continental region of Alsace over recent decades (Dubois et al. 1991; Dronneau 2014), but trends are unclear for the coastal region of Hauts‐de‐France. Our three main study objectives were to: (1) identify the primary causes of nest failure and estimate rates of nest survival in the two regions; (2) evaluate the potential effects of daily weather conditions on nest survival rates; and (3) assess whether marking the nests with our field equipment resulted in any difference in nest survival rates or causes of nest losses as compared to nests that were left unmarked. To address our objectives, we monitored nests with nest cameras and through repeated visits, evaluated local microclimatic conditions through modelling, and constructed nest survival models incorporating the effects of local environmental conditions.

If the factors driving the reproductive success of lapwings at our field sites are similar to those of other waders nesting in agricultural systems, we predicted: (1) the majority of nest losses would be caused by a combination of depredation and agricultural operations, with a lower rate of nest survival in Alsace, where local populations have shown a decline (Dubois et al. 1991, Dronneau 2014); (2) a lower rate of nest survival on days without rain (Boyle et al. 2020) and days with high temperatures (Carroll et al. 2018); (3) differences in the cause of loss for nest failures between nests equipped with cameras versus control nests. We predicted that predators might either learn to find and depredate nests with markings and cameras or conversely, might be wary of novel objects encountered in their habitat (Winder et al. 2016). We thus expected to find an impact of monitoring on nest survival rates, but the difference could be either positive or negative.

## 2 Materials and Methods

### 2.1 Field Sites

**FIGURE 1:**
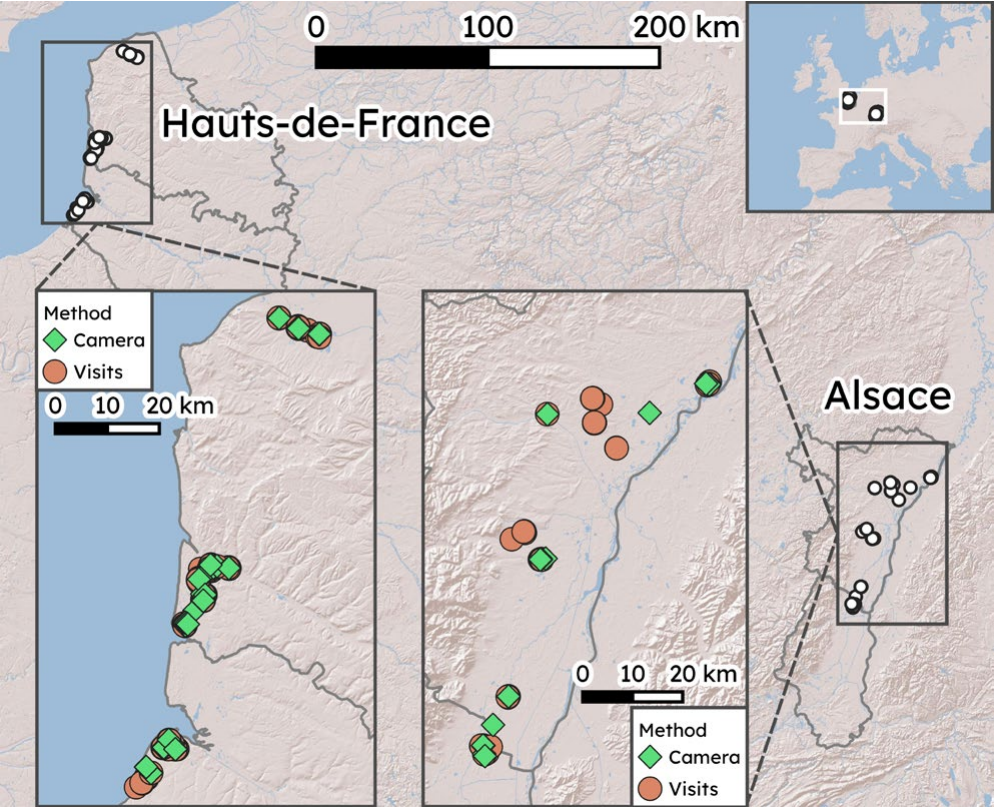
Locations and monitoring methods for Northern Lapwing nests in two regions of France, 2021–2022 (*n* = 145). Green diamonds and orange circles indicate whether the nest was monitored by camera or by visits only. Map image tiles from the ESRI shaded relief map.

We conducted regional surveys for nesting pairs of Northern Lapwings during the breeding seasons of 2021 and 2022 in two regions of France: the western region of Hauts‐de‐France and the eastern region of Alsace (Figure 1). Surveys in Hauts‐de‐France occurred primarily in coastal flatlands, whereas the Alsatian surveys occurred primarily in the Rhine river valley. At a departmental level, the Hauts‐de‐France region consisted of the departments of Somme and Pas‐de‐Calais, while the Alsace region included the departments of Bas‐Rhin and Haut‐Rhin.

Reports of agricultural land‐use in 2020 indicated that land use differed between the two regions (Table 1; Agreste 2020). The main types of agricultural land use in Hauts‐de‐France included non‐maize cereal crops (45.6%), permanent grasslands (12.3%), industrial crops such as sugar beets (9.8%), potatoes (7.8%), alfalfa, clover, and other fodder crops (7.2%), oil crops (5.2%), and low coverage of maize and other crop types (≤ 2%). By contrast, the dominant types of agriculture in Alsace included maize (36.5%), permanent grasslands (24.0%), non‐maize cereals (17.7%), and vineyards (5.4%), with low coverage in other land types. Legumes, temporary grasslands, and fallow land were present in both regions, but the areal coverage was < 3% (Agreste 2020).

**TABLE 1:** Percentages of total agricultural area dedicated to different crops or types of agriculture in 2020 in the two regions of this study.

Agricultural land use	Percentage of total agricultural area	
	Hauts‐de‐France (Pas‐de‐Calais & Somme)	Alsace (Bas‐Rhin & Haut‐Rhin)
Cereals (excluding maize)*	45.6%	17.7%
Maize	2.0%	36.5%
Oil crops	5.2%	4.2%
Fibre crops	3.4%	0.0%
Industrial crops (including sugar beets)	9.8%	2.0%
Legumes	1.0%	0.1%
Fodder, alfalfa, and clover*	7.2%	4.3%
Potatoes	7.8%	0.4%
Open‐air vegetables and flowers*	3.3%	0.9%
Temporary grasslands (< 5 years old)	1.3%	2.8%
Permanent grasslands	12.3%	24.0%
Permaculture (including vineyards)*	0.1%	5.4%
Fallow land	0.6%	1.5%
Other agricultural land	0.3%	0.1%
Total agricultural land area (hectares)	917,501 ha	331,658 ha

*Note:* Adapted from publicly accessible data provided by the French government in the context of the European Common Agricultural Policy (Agreste 2020). Asterisks ‘*’ indicate where the source data has been grouped by crop type.

Nests in Hauts‐de‐France were found in a 596 km^2^ area (Minimum Convex Polygon (MCP) of nests found), whereas nests in Alsace were located in a 1111 km^2^ area (MCP of nests found). The two regions are 500 km apart and separated by the Vosges mountain range. Surveys took place between 6th of April and 20th of July in 2021 (106 days), and between 15th of February and 29th of June in 2022 (135 days). The field study was conducted during the years of the Covid‐19 global pandemic, which affected the logistics of site access, travel, and field activities.

### 2.2 Nest Monitoring

Lapwings were located by observing birds with binoculars and a spotting scope, as well as naked‐eye observations and auditory cues. Birds' breeding status was assessed based on their behaviour: territorial males performing display flights and ground displays were used to locate the nesting sites (Rinkel 1940).

Having approximately located habitats containing territories of breeding males, we then searched for lapwing nests within the territories. Observations of incubating adults provided the main indication of potential nest location, except at three wetland sites, where searching along transects instead provided locations for 25 nests. Potential nest locations were approached on foot for confirmation. At each identified nest, we recorded the GPS coordinates and the number of eggs in the clutch. Monitored nests were then visited on a weekly basis to determine nest fate. Where permission to place equipment was obtained from landowners, nest cameras (Victure HC300 Trail Camera) were attached to 1 m high pine wood poles, with a 6 cm diameter, hammered into the ground between 10 and 25 cm (depending on substrate hardness and stability) at a 2 m distance from the nests. A set of anti‐pigeon spikes was affixed to the top of each pole to discourage corvids or raptors from attempting to perch on the camera poles.

Nest cameras captured still images at a resolution of 5200 × 3900 pixels and then 30 s videos of 1920 × 1080 pixels at a 60° viewing angle after being triggered by a motion sensor with a 90° detection angle. The manufacturer indicated a 0.3 s trigger speed for this model, which we considered adequate for the photo function. However, the videos were likely recorded at a greater delay based on observed posture differences between still images and the first frames of video footage. At night, the cameras used an integrated set of infrared LEDs, operating outside the range of human vision, to illuminate the area directly in front of the camera upon activation.

Photos and videos were then used to verify nest fates. If footage was unavailable, we relied on direct observations of chicks around the nest cup as well as indirect assessments of nest fate. Parents remove large pieces of eggshells at hatching, but clutches were considered as successfully hatched if small eggshell fragments were found in the nest lining (Mabee et al. 2006; Thorup 2022). If evidence at the nest site indicated at least one egg hatched and produced a chick, a nest was considered to be successful.

A nest was considered to have failed if: (1) direct evidence indicated failure, for example through the continued presence of a full clutch beyond a 30‐day period of observation, (2) the eggs disappeared without further clues to their fate before the expected hatching date as determined from egg flotation (Van Paassen et al. 1984), (3) the soil around the nest site was disturbed by farming equipment and the nest cup could not be relocated, or (4) the nest site was damaged by flooding. Clutches of lapwings were monitored until the outcome of the incubation attempt could be established as either failed or hatched.

### 2.3 Weather Parameters

To obtain information on local weather conditions, we used the R package *NichemapR* (Kearney and Porter 2017), which uses a combination of historic data from satellites and ground‐based stations to estimate weather conditions for any given location on Earth. The estimates are provided on an hourly basis, using weather parameters interpolated from 6‐hourly source data. We relied on the ERA5 dataset for our microclimate modelling (Hersbach et al. 2020), as implemented through the function *micro_era5* of the R package *NichemapR* (Klinges et al. 2022), which relies on functions from the package *microclima* (Maclean et al. 2019).

We estimated weather conditions for every nest for the duration of the breeding season, using locations based on the GPS coordinates. For our analysis of nest survival, we used five different variables: (i) air temperature at 1 cm above soil surface, (ii) soil surface temperature, (iii) wind speed at 1 cm above surface, (iv) daily rainfall, and (v) solar irradiance. The last two variables were not hourly, but extracted as daily totals. We extracted daily means, maxima, and minima for each variable where available, for use as daily covariates in a nest survival model.

### 2.4 Statistical Analysis

All statistical analyses were performed in an *R* environment (version 4.2.2, R Core Team 2022). We compared the available environmental variables between our two regions by modelling each variable in a linear model featuring the region and a linear effect of day of the year as fixed variables and the date as a random variable.

#### 2.4.1 Incubation Failure Causes

To determine whether there were any differences in nest fates between regions and monitoring methods, we used Fisher's Exact Tests from the base R package *stats*. We ran the analysis of the region for the full dataset and again for the subset of nests that were monitored by camera. The camera dataset provides direct information on failure causes, while the causes of failures for ‘visits only’ nests where chicks were not encountered directly in the nest cup were inferred from signs at the nest site. We assessed any determined differences on a category‐by‐category basis using Holm‐Bonferroni‐corrected post hoc tests using the function *row_wise_fisher_test* from the package *rstatix* (Kassambara 2023).

To investigate whether the timing of different nest fates was distributed equally over the duration of the breeding season, potential differences in this timing were estimated with a Gaussian linear regression from the base R package *stats*, modelling the day of the year the clutch was last observed active as a function of the eventual nest fate. We calculated post hoc contrasts using the package *emmeans* (Lenth and Piaskowski 2026). We excluded flooding from this analysis because it was a cause of failure for only two nests in our dataset.

#### 2.4.2 Nest Survival Modelling

For our analyses of daily nest survival, we used a nest survival procedure for known fate data (Dinsmore et al. 2002), using the R package *Rmark* as an interface to MARK (Laake 2013). The nest survival procedure allowed us to model daily survival rates (DSR) of nests based on the number of ‘exposure days’ over which nests were monitored and at risk of failure. Nests were considered at risk and included in our analysis from the day they were discovered with at least one egg in the nest cup (*i*), regardless of whether the clutch had been completed or incubation had begun. We then recorded the last day on which the nest was known to be active (*j*), and the day on which the nesting attempt was confirmed to be completed (*k*). In the case of a successful nesting attempt, *j* and *k* were the same day. In the case of unsuccessful nests, *j* was lower than *k*, and the two dates bracketed the period of possible nest failure.

The median number of days between nest visits was 7 (IQR: 6–7), a rate comparable to the data presented by Dinsmore et al. (2002). Nests monitored by cameras provided more accurate information on the timing of nest outcomes. While the precision of knowledge about nest fates by cameras could in some cases be expressed in minutes, we nonetheless treated these as accurate only to the level of daily visits, such that a nest survived a day if it survived until midnight.

We also included environmental variables for each individual nest, which could be dynamic and different for each day of the breeding season, like in the case of daily rainfall, or static over the breeding season, like region or year.

Nest survival modelling proceeded in four steps: variable selection, model building, model selection, and parameter estimation. During variable selection, we used pairwise comparisons to test for collinearities among available microclimate variables such as mean soil temperature, maximum soil temperature, and mean wind speed. We considered relationships as correlated strongly enough to warrant exclusion at r values greater than |0.65| (Dormann et al. 2013). In those cases, we included the variable that we expected to be most directly ecologically relevant to nest survival.

We used a model‐building procedure where we tested every model that was nested within the top‐level (global) model. We defined the top‐level model as the model predicting daily survival rates (S) from all grouping variables and the selected environmental variables (Formula 1), as well as potentially ecologically relevant interaction effects. We considered five grouping variables: habitat type (crop fields/other), year (2021/2022), region (Alsace/Hauts‐de‐France), clutch size (< 4/4 eggs), and monitoring method (camera/visits). Because the duration of exposure to adverse conditions can be a strong influence on physiological outcomes (Hainsworth et al. 1968), we opted to include 1‐day lagged variables for the main environmental variables of ground temperature and wind speed. Inclusion of a 1‐day lag tested for differences between long periods of adverse conditions and brief exposures while limiting the extended computation time that a more complex set of lagged variables would require. Seasonal variation in daily survival was included as ‘days into the season’, equivalent to a ‘day of year’ variable, where the first occasion was the day that the first nest with eggs was discovered.

In addition to the main effects, we considered five possible interactions among the factors that were relevant to our particular study system. We expected there might be an interaction between mean soil temperature and mean wind speed if the convective cooling effect of higher wind speeds was more important for birds experiencing higher soil temperatures, and the opposite for lower temperatures (Bakken et al. 2002; Reid et al. 2002).

We considered an interaction effect between region and wind speed because we expected there might be overall climatic differences between the two different regions. Our exploratory analysis indicated that the correlation between wind speed and soil temperature was different for the two regions.

We considered an interaction between the monitoring method and region because nests without a camera might be exposed to different baseline risks if predator communities or farming practices differed between regions.

Last, we considered the interactions between the two main environmental variables and their 1‐day lagged variables. We considered that the interaction between the 2 days' mean wind speeds and temperatures might be the best way to incorporate the lagged variables in the model to allow them to produce a distinction between long‐term weather conditions and single gusty/hot days, and so included them as a pair of interaction effects. Our global model for nest survival was given by the following expression:

(1) $$\begin{array}{c} \text{Nest survival}\sim \text{Habitat type}+\text{Year}+\text{Eggs in clutch}+\text{Daily rainfall}\\ +\text{Total solar irradiance}+{\text{Mean daily soil temperature}}^{*}\;\text{Mean daily wind speed}\\ +{\text{Region}}^{*}\;\text{Mean daily wind speed}+\text{Previous}\;{\mathrm{day}}^{'}s\;{\text{mean daily soil temperature}}^{*}\;\text{Mean daily soil temperature}\\ +\text{Previous}\;{\mathrm{day}}^{'}s\;{\text{mean daily wind speed}}^{*}\;\text{Mean daily wind speed}\\ +{\text{Region}}^{*}\;\text{Monitoring method}+\text{Days into the season} \end{array}$$

Model selection was based on the differences in AICc scores among the candidate models (‘Akaike Information Criterion*’*, Sugiura 1978, Akaike 1998). Models were not considered if a combination of grouping variables included in the model was significantly unbalanced, as determined by a chi‐square test, or resulted in a group with ≤ 5 nests. In the special case where two models differed by a single parameter (Arnold 2010), we tested whether covariates might be uninformative based on the 95% Confidence Intervals (CI) of the parameter estimate on the logit scale. We considered parameters as weakly predictive if the CI of the estimated effect overlapped 0. We excluded models where all variables' CIs overlapped 0, but not those with at least one significant predictor. The remaining models were ranked by their AICc score (Table 2).

**TABLE 2:** Estimates of model coefficients for all models ≤ 2 AICc of the best‐fit model, and the null or intercept‐only model used to describe Northern Lapwing nest survival.

Model	Intercept	Camera	Region	Soil temperature	Wind Speed	Previous day's temperature	Previous day's wind speed	Rain	DOS	Solar irradiance	Camera: region	Region: wind	Wind speed: previous day's wind speed	K	logLik	AICc	ΔAICc	Weight
Top fitted	−1.013	1.931*	1.700*	NA	1.905	NA	7.732*	NA	NA	NA	−1.708*	NA	−5.741*	7	−165.626	345.309	0	0.047
2	−0.918	1.965*	0.347	NA	0.825	NA	9.438*	NA	NA	NA	−1.737*	3.020	−8.119*	8	−164.935	345.942	0.634	0.035
3	−1.043	1.889*	1.655*	NA	1.639	NA	7.839*	0.060	NA	NA	−1.658*	NA	−5.597*	8	−165.126	346.326	1.017	0.028
4	−2.093	1.894*	1.604*	0.037	2.694	NA	8.391*	NA	NA	NA	−1.657*	NA	−6.543*	8	−165.128	346.329	1.020	0.028
5	−1.833	1.877*	1.597*	NA	2.200	0.031	8.403*	NA	NA	NA	−1.640*	NA	−6.159*	8	−165.207	346.488	1.179	0.026
6	−0.945	1.914*	0.284*	NA	0.600	NA	9.542*	0.060	NA	NA	−1.679*	3.077	−8.089*	9	−164.421	346.933	1.624	0.021
7	−1.120	1.884*	1.649*	NA	1.960	NA	7.780*	NA	0.003	NA	−1.656*	NA	−5.805*	8	−165.573	347.219	1.910	0.018
8	−2.152	1.840*	1.553*	0.038	2.455	NA	8.510*	0.062	NA	NA	−1.595*	NA	−6.434*	9	−164.579	347.249	1.940	0.018
9	−0.860	1.925*	1.701*	NA	1.826	NA	7.714*	NA	NA	−0.00002	−1.701*	NA	−5.666*	8	−165.613	347.299	1.990	0.018
Null model	3.678*	NA	NA	NA	NA	NA	NA	NA	NA	NA	NA	NA	NA	1	−197.431	396.863	51.554	< 0.001

*Note:* Values in columns 2 through 14 indicate variables included in the models, in which case the slope estimate is provided on the logit scale, or NA in the case of its absence. Bolded values followed by an asterisk (*) indicate variables for which the 95% confidence interval does not include 0 (‘Informative variables’). Variables include: Whether a camera was placed at the nest (Camera), whether the region in which the nest was found was Alsace or Hauts‐de‐France (Region), soil surface temperature in degrees Celsius (Soil Temperature), wind speed at 1 cm above surface in m/s (Wind Speed), the mean soil temperature of the day preceding the considered day in degrees Celsius (Previous day's Temperature), the mean wind speed at 1 cm above surface of the day preceding the considered day in m/s (Previous Day's Wind Speed), daily rainfall in mm (Rain), day of the season in days since the 6th of April of that year (DOS), solar irradiance at the surface in Wm^−2^ (Solar Irradiance), the interaction effect between Camera and Region, the interaction effect between region and wind speed, and the interaction effect between wind speed and the previous day's wind speed. The final five columns indicate the number of parameters (*k*), log‐likelihood of the candidate model (logLik), AICc score (AICc), difference in AICc score as compared to the minimum AICc model (ΔAICc), and model weight relative to all proposed models (Weight). None of the top model candidates included the categorical variables ‘Number of eggs in the nest’, ‘Habitat type’, or ‘Year’, or the interaction effect between mean daily soil temperature and the previous day's mean daily soil temperature.

Using our best‐fit model, we estimated the probability of surviving an average incubation period (27 days, Larsen et al. 2003) for different combinations of region, year, and monitoring method. We calculated the probability of surviving any day during which nests were monitored, and created 1000 bootstrap replicates based on our weather data. The probability of a hypothetical nest surviving 27 consecutive days was then calculated for every starting day between the beginning and end of our breeding seasons, less 26 days at the end. The calculations provided a survival estimate that took into account varying daily survival rates (Weiser 2021). We corrected for slight discrepancies by calculating the difference between the mean of the bootstrap distribution and the expected mean based on mean DSR, and subtracted the difference from all of the bootstrap replicates to re‐center the mean. Our 95% CIs for the estimated mean probability of surviving incubation were then taken as the 2.5% – 97.5% quantiles of the corrected bootstrap distributions.

## 3 Results

### 3.1 Nest Monitoring

Of 182 nests found with eggs, we included 145 nests with complete information in our nest survival analysis. For 37 remaining nests (20% of nests), we could not accurately determine nest fate either due to restrictions on land access during the COVID‐19 pandemic, or in some cases, due to the lack of signs at the nest site to determine the nest fates reliably. Of our 145 nests, 37 were located in Alsace (20 with a camera and 17 monitored by visits only) and 108 were located in Hauts‐de‐France (72 with a camera and 36 monitored by visits only). Of the nests in Hauts‐de‐France, 30 were found in the 2021 breeding season (26 with a camera and 4 with visits only) and 78 were found in the 2022 breeding season (46 with a camera and 32 with visits only). Of the nests in Alsace, 10 were found in the 2021 breeding season (6 with a camera and 4 with visits only) and 27 were found in the 2022 breeding season (14 with a camera and 13 with visits only). Over both regions and years, 92 nests were monitored by camera and 53 nests were monitored by visits only. The median monitoring duration for all nests was 14 days (interquartile range (IQR): 8–22 days).

All nests were used to determine habitat associations of breeding lapwings. Lapwing nests found on agricultural fields were primarily located in maize fields (74 in Hauts‐de‐France, 33 in Alsace). In addition, eight nests were found on spring‐sown cereal (7 in Hauts‐de‐France, 1 in Alsace), seven on pastures that were recently grazed or about to be grazed (all in Hauts‐de‐France), five nests each on beet and potato fields (all in Hauts‐de‐France), four on soybeans in Alsace, two on peas in Hauts‐de‐France, two on fallow fields in Alsace, two on flax fields in Hauts‐de‐France, and two on a chicory field in Hauts‐de‐France. Last, one nest was found on a market garden in Hauts‐de‐France following a conversation with the farmer. Ten nests were found on tillage before sowing in Alsace, but none in Hauts‐de‐France.

### 3.2 Environmental Differences

We found small differences in environmental variables between the two regions (Table 3). Air temperatures were 1°C–3°C higher in Alsace than Hauts‐de‐France. The same was true for soil surface temperatures. Conversely, minimum, maximum, and mean wind speeds were 0.2–0.4 m/s higher at Hauts‐de‐France than Alsace. Temperature ranges within single days were 2.4°C greater in Alsace, while wind speed ranges were 0.17 m/s greater in Hauts‐de‐France. Daily rainfall was 0.4 mm greater at Alsace, and 31% of all nest‐days were dry. Mean solar irradiance was 6 W/m^2^ higher in Hauts‐de‐France, but maximum solar irradiance did not differ between regions.

**TABLE 3:** Estimated marginal means of environmental variables for each exposure day of every Northern Lapwing nest in our dataset, grouped by region (*n* = 2087 nest‐days, 367 in Alsace and 1720 in Hauts‐de‐France).

Model	Estimate Alsace (mean ± SE)	Estimate Hauts‐de‐France (mean ± SE)	*p*‐value in linear mixed model	Magnitude of difference
Mean air temperature (°C)	15.8 ± 0.3	13.9 + 0.27	**< 0.001**	+1.9°C
Maximum air temperature (°C)	24.6 ± 0.6	21.3 +0.6	**< 0.001**	+3.3°C
Minimum air temperature (°C)	8.7 ± 0.2	7.7 + 0.2	**< 0.001**	+1.0°C
Mean soil temperature (°C)	17.2 ± 0.4	15.5 + 0.3	**< 0.001**	+1.7°C
Maximum soil temperature (°C)	30.7 ± 0.9	27.4 + 0.9	**< 0.001**	+3.3°C
Minimum soil temperature (°C)	8.1 ± 0.2	7.3 + 0.2	**< 0.001**	+0.8°C
Mean wind speed (m/s)	0.3 ± 0.02	0.6 + 0.02	**< 0.001**	+0.3 m/s
Maximum wind speed (m/s)	0.5 ± 0.02	0.9 + 0.03	**< 0.001**	−0.4 m/s
Minimum wind speed (m/s)	0.2 ± 0.02	0.4 + 0.02	**< 0.001**	−0.2 m/s
Air temperature range (°C)	16.0 ± 0.6	13.6 + 0.6	**< 0.001**	+2.4°C
Soil temperature range (°C)	22.6 ± 1.0	20.2 + 0.9	**< 0.001**	+2.4°C
Wind speed range	0.4 ± 0.02	0.5 ± 0.02	**< 0.001**	−0.1 m/s
Rain (mm)	2.6 ± 0.3	2.2 ± 0.3	**0.015**	+0.4 mm
Mean solar irradiance (W/m^2^)	217 ± 5.6	223 ± 5.3	**0.007**	−6 W/m^2^
Maximum solar irradiance (W/m^2^)	668 ± 14.9	667 ± 14.1	0.802	+1 W/m^2^

*Note:* Air temperature and wind speed variables were modelled at 1 cm above soil surface. *p*‐values indicate the estimated significance of the independent effect of region in a linear model predicting the environmental variable (*x*) as a function of the region (*r*) and day of the year (DOY), controlling for the random effect of date (date) in the form: *x* ~ *r* + DOY + (1 | date). Bolded values indicate *p* < 0.05.

### 3.3 Causes of Nest Failure

**FIGURE 2:**
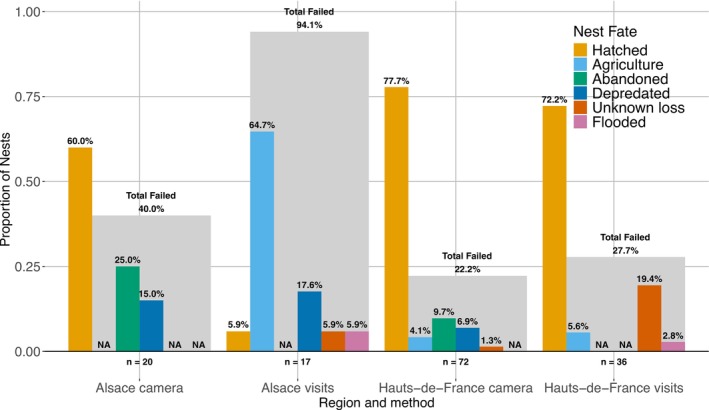
Observed proportions of Northern Lapwing nest fates for each combination of region and nest monitoring method in two regions of France, 2021–2022 (*n* = 145). Nests for which nest fate could not be clearly determined are not shown (*n* = 39). The shaded grey bars indicate the total proportion of unsuccessful nests.

The observed nest fates differed between the two study regions (Fisher's Exact Test, *n* = 145, *p* < 0.001, Table 4, Figure 2). Alsatian nests were less likely to hatch than nests in Hauts‐de‐France (35% of nests vs. 76% of nests, odds ratio 0.174, Pairwise Fisher's Test with HB‐correction, *n* = 145, adjusted *p* < 0.001). In addition, nests in Alsace were more likely to be lost to agricultural operations (30% of nests vs. 5% of nests, odds ratio 8.54, Pairwise Fisher's Test with HB‐correction, *n* = 145, adjusted *p* < 0.001). When we considered only camera‐monitored nests, we found no difference in nest fates between regions (Fisher's Exact Test, *n* = 92, *p* = 0.198).

**TABLE 4:** Pairwise comparisons of nest fate for Northern Lapwings nesting in two regions of France, and under two different monitoring methods, for the same 145 nests in the breeding seasons of 2021 and 2022.

Comparison between regions	Hatched	Lost to agriculture	Abandoned	Flooded	Depredated	Unknown
Alsace (*n* = 37)	**13 (35%)**	**11 (30%)**	5 (14%)	1 (3%)	6 (16%)	1 (3%)
Hauts‐de‐France (*n* = 108)	**82 (76%)**	**5 (5%)**	7 (6%)	1 (1%)	5 (5%)	8 (7%)
Total (*n* = 145)	95 (66%)	16 (11%)	12 (8%)	2 (1%)	11 (8%)	9 (6%)

Comparison between methods	Hatched	Lost to agriculture	Abandoned	Flooded	Depredated & Unknown
Visits only (*n* = 53)	**27 (50%)**	**13 (25%)**	**0 (0%)**	2 (4%)	11 (21%)
Camera (*n* = 92)	**68 (74%)**	**3 (3%)**	**12 (13%)**	0 (0%)	9 (10%)
Total (*n* = 145)	95 (66%)	16 (11%)	12 (8%)	2 (1%)	20 (14%)

*Note:* Significant differences are indicated in bold. Percentages are displayed based on row totals.

Predators were identified from camera footage at nine nests during two field seasons: five nests were depredated by red foxes, two by beech martens ( *Martes foina* ), one by a European badger ( *Meles meles* ), and one by carrion crows ( *Corvus corone* ). Two additional nests were clearly depredated based on eggshell remains, but the predator could not be identified. The nine nests lost to unknown causes were likely depredated because their clutches disappeared from the nest cup while the surrounding soil was undisturbed. 12 nests were abandoned, and two nests were lost to flooding (Figure 2).

Under the assumption that disappearing clutches were likely depredated, we analysed the occurrence of nest outcomes between our two monitoring methods. Nest fates were not equally distributed between methods (Fisher's Exact Test, *n* = 145, *p* < 0.001, Table 4). The only nests confirmed to have been abandoned were under camera monitoring (odds ratio 0, Pairwise Fisher's Test with HB‐correction, *n* = 145, adjusted *p* = 0.016), whereas nests were more likely to have been destroyed by agriculture if no camera was present (odds ratio 9.48, Pairwise Fisher's Test with HB‐correction, *n* = 145, adjusted *p* < 0.001). Further, nests were less likely to hatch if no camera was present (odds ratio 0.369, Pairwise Fisher's Test with HB‐correction, *n* = 145, adjusted *p* = 0.020). No difference was found for the combination of depredation and unknown failures (odds ratio 2.40, Pairwise Fisher's Test with HB‐correction, *n* = 145, adjusted *p* = 0.163).

Based on available camera footage, abandonments were determined to have taken place directly after the placement of the camera in six out of 12 cases. In one case, a camera was placed at an incomplete clutch, which was not completed (no cameras were placed at clearly incomplete clutches thereafter). In the remaining five cases of abandonment, incubation was confirmed to have continued after camera placement.

**FIGURE 3:**
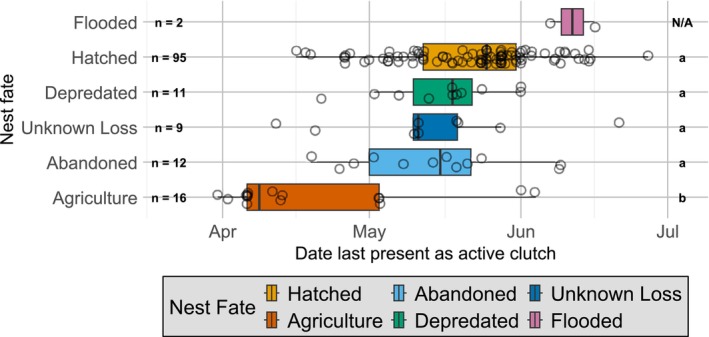
Seasonal timing of nest fate for Northern Lapwings in two regions of France during the breeding seasons 2021–2022 (*n* = 145). Points indicate individual clutches and are jittered vertically to avoid overlap.

We found that the median timing of failure for nests lost to agriculture was in early‐April, 24–33 days earlier in the season as compared to other nest fates (Tukey's HSD, *p* < 0.003 for four pairwise comparisons, Figure 3). The other nest fates did not occur at significantly different times from each other, with most successes and failures in mid‐ to late‐May (Tukey's HSD, *p* > 0.432 for six pairwise comparisons, Figure 3). Two nests were lost to floods during the same year, in June.

### 3.4 Nest Survival

**FIGURE 4:**
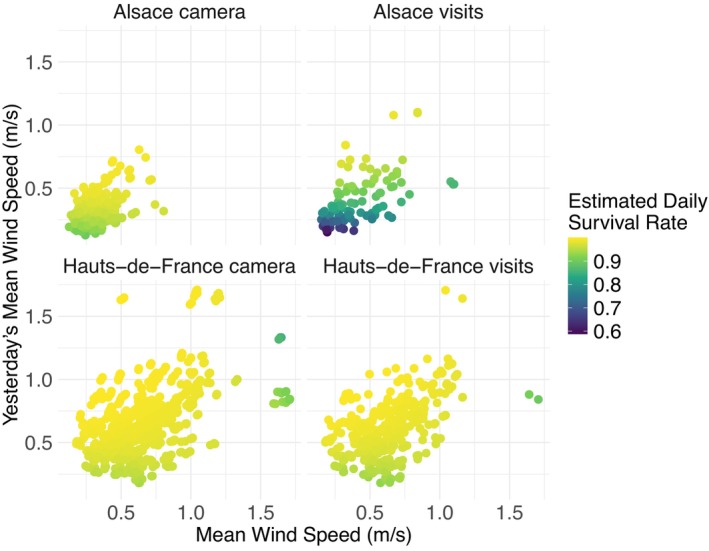
Estimates of the daily survival rate of Northern Lapwing nests as a function of the region, nest monitoring method, local mean daily wind speed, and mean daily wind speed of the previous day. *n* = 2087 exposure days: 246 Alsace camera, 121 Alsace visits, 1112 Hauts‐de‐France camera, 608 Hauts‐de‐France visits.

Daily nest survival rates were best explained by a combination of six factors: (1) the region in which the nest is present; (2) the monitoring method; (3) an interaction effect between monitoring method and region; (4) the mean wind speed on a given day; (5) the mean wind speed on the day prior; and (6) the interaction effect between wind speed on the day itself and the day prior (Tables 2 and 5, Figure 4).

**TABLE 5:** Estimated effects, standard errors (SE), lower‐ and upper 95% confidence intervals (CI) for each variable of the best‐performing model: Daily nest survival ~ Region * Monitoring method + Mean daily wind speed * Previous day's mean daily wind speed.

Variable	Estimate	SE	Lower CI	Upper CI
Intercept	−1.013	0.915	−2.807	0.781
Region: Hauts‐de‐France	1.700	0.565	**0.591**	**2.808**
Monitoring method: camera	1.931	0.470	**1.009**	**2.852**
Mean wind speed	1.905	1.900	−1.820	5.630
Previous day's mean wind speed	7.732	2.097	**3.621**	**11.843**
Hauts‐de‐France + camera	−1.708	0.626	**−2.933**	**−0.481**
Wind speed: previous day's wind speed	−5.741	2.533	**−10.706**	**−0.777**

*Note:* All numbers are presented on the logit scale. Confidence intervals are bolded where they do not overlap 0. The effect of wind speed, for which the CI overlaps 0, is considered uninformative for estimating nest survival rate when considered separately but contributes positively to the accuracy of the entire model as compared to a model that excludes it.

We used the variable effect estimates from the top‐level model to estimate daily survival rates for recorded nest‐days. The best‐fit model showed that daily nest survival rates were high in the region of Hauts‐de‐France, were only moderately reduced at low wind speeds, and were higher with camera monitoring (Without camera: median DSR = 0.987, IQR 0.978–0.991, *n* = 608 exposure days. With camera: median DSR = 0.989, IQR 0.982–0.994, *n* = 1112 exposure days, Table 6, Figure 4). Daily nest survival rates were high in Alsace for high wind speeds, but were reduced by low wind speeds or factors related to low wind speed, especially when cameras were absent (Without camera: median DSR = 0.842, IQR 0.777–0.924, *n* = 121 exposure days. With camera: median DSR = 0.967, IQR 0.949–0.981, *n* = 246 exposure days, Table 6, Figure 4).

**TABLE 6:** Median predicted nest survival rates for all Northern Lapwing nests over their exposure days, and associated interquartile ranges. For each region and monitoring method grouping, the average of the mean daily wind speed of all nests is provided.

Region	Monitoring method	Median daily survival rate	Interquartile range	Mean of mean daily wind speed (m/s)	Exposure days
Alsace	Camera	0.967	0.949–0.981	0.335	246
Alsace	Visits	0.842	0.777–0.924	0.426	121
Hauts‐de‐France	Camera	0.989	0.982–0.994	0.652	1112
Hauts‐de‐France	Visits	0.987	0.978–0.991	0.624	608

Deployment of a camera at a nest was associated with an increase in daily nest survival rates (Table 6). We had predicted that equipment at the nest might negatively affect nest survival if predators were attracted to the nest site, but the mean daily survival rate estimate for camera‐equipped nests in Hauts‐de‐France was estimated to be 0.003 higher per day than for nests without a camera. The slight difference resulted in a 17% reduction in the estimated daily probability of failure (complement of daily survival rate), because of the high survival rates under both monitoring methods. The mean daily survival rate estimate for camera‐equipped nests in Alsace was estimated to be 0.13 per day higher than for nests without equipment. The difference resulted in a 78% reduction in the estimated daily probability of failure.

The mean estimated probabilities to survive a full incubation period were greater at the coastal sites of Hauts‐de‐France than at the inland sites of Alsace. Lapwing nests in Hauts‐de‐France (1000 bootstrapped replicates each for 173 days over 2 years) had a mean estimated probability to survive incubation of 0.656 (bootstrap SE = 0.051) when monitored by a camera and a probability of 0.592 (bootstrap SE = 0.057) when not monitored by a camera. By contrast, lapwing nests in Alsace (1000 bootstrapped replicates each for 151 days over 2 years) had a mean estimated probability to survive incubation of 0.428 (bootstrap SE = 0.035) when monitored by camera and a probability of 0.007 (bootstrap SE = 0.003) when not monitored by camera.

The grouping variables of year, habitat type, and number of eggs in the clutch were not included in any of the candidate models that were separated by ≤ 2 AICc score from the best‐fit model, nor was the interaction effect between mean daily soil temperature and the previous day's mean daily soil temperature (Table 2). The numeric variables of daily rainfall, mean daily soil temperature, solar irradiance, day of the year, and the interaction effect between region and wind speed were included in some highly ranked candidate models, but not the top‐fitted model (Table 2).

## 4 Discussion

In our 2‐year field study, we investigated factors affecting the nesting success of Northern Lapwings at coastal and inland breeding sites in France, where the majority of lapwings were found breeding on crop fields. Our new information on nest survival and causes of failure will assist conservation efforts for lapwings in France, where the species has been in a nationwide decline (Joyeux et al. 2022), and provides insights that can be generalised to other ground‐nesting birds in agricultural habitats. Monitoring lapwing nests during the incubation stage of the reproductive cycle provided three results for the demography of the species. First, nest survival differed between regions and may result in regional source‐sink dynamics or local extinctions in France if the observed differences persist into the future. Second, local weather conditions were related to nest survival rates, with the lowest survival under low mean wind speed conditions. Last, deploying cameras at the nests resulted in a net‐positive effect on nest survival rates, potentially due to a reduction in losses to agriculture combined with no net difference in nest depredation.

### 4.1 Nest Fates

Average rates of nest survival for breeding lapwings in Western Europe have been reported as 43% before 1980, and 32% between 1996 and 2006 (Roodbergen et al. 2012). More recent figures have been reported in the range of 32%–85% (Eglington et al. 2009, Bodey et al. 2011, Kamp et al. 2015, Bertholdt et al. 2017). A majority of lapwing nests monitored at our field sites in two regions of France were observed to hatch and produce young (66%), a favourable rate compared to the European average. However, we also found differences in nesting success between the two study regions: Lapwing nests in Alsace were less likely to hatch and more likely to be lost to agricultural operations than nests in Hauts‐de‐France, resulting in a 41 percentage‐point difference in observed hatching success. Differences in nesting outcomes between field sites have been previously reported elsewhere in Europe (Seymour et al. 2003; Teunissen et al. 2008). Our study regions included summaries of nest data over a large area but inter‐site differences in France are likely to be important determinants of nesting outcome, as they are in other European countries.

Predation is often the main cause of reproductive losses for breeding lapwings in Europe (Teunissen et al. 2006; Bellebaum and Bock 2009; Eglington et al. 2009), with nest depredation rates reported in the range of 17%–61% (Schifferli et al. 2006; MacDonald and Bolton 2008a; Bodey et al. 2011; Düttmann et al. 2018; Korner et al. 2024). Agricultural operations can be responsible for between 1% and 71% of location‐specific nest outcomes, with the most severe losses occurring where lapwings breed on intensively farmed sites without nest protection measures, and the fewest losses where an intensive nest marking effort has been implemented by researchers or volunteers (Berg et al. 2002; Teunissen et al. 2006; Sheldon et al. 2007; Bellebaum and Bock 2009; Korner et al. 2024).

We predicted that France would not be exceptional in this regard, with at least 50% of nest failures likely to be caused by the combination of depredation and agricultural operations. Our prediction was confirmed because a majority of nest losses at our sites were due to either confirmed depredations or agricultural operations (54%). If we include the nests that failed due to unknown causes, the figure would increase to 72% of observed nest failures.

Unexpectedly, we found no differences in rates of depredation between regions or between monitoring methods, potentially related to the relatively low number of observed depredation events. Even if we consider the unknown losses to have been caused by depredation, our total apparent losses to predation are comparatively low at 14% of nests, which is comparable to previous studies with significant nest protection efforts (20% in Schifferli et al. 2006 including also the abandoned and flooded nests in the total, 16.5% in Korner et al. 2024). Most depredation events at our nests were attributed to mammalian predators where a species‐level determination was possible. Our results are consistent with previous work on causes of nest failure for waders elsewhere in continental Europe, where nest losses are frequently caused by mammalian predators (Teunissen et al. 2008; MacDonald and Bolton 2008b).

In contrast to the relatively low depredation rates, abandonment rates for nests equipped with cameras (25.0% and 9.7% of nests with known fates in Alsace and Hauts‐de‐France, respectively, 13% of all camera‐equipped nests) were above rates previously reported for lapwing nests with nest cameras or nest cages placed in close proximity (2.5% and 8.1%, Bolton, Butcher, et al. 2007; Isaksson et al. 2007). In another study, a comparable abandonment rate of 15.3% followed from a combination of high nest visitation rates at 1–2 day intervals, bamboo stick marking, fencing, and ringing of chicks (Korner et al. 2024). In our own study, we could not establish whether differences in monitoring methods, regional conditions, or other factors were responsible for observed rates of abandonment, because we had difficulties identifying abandonment at nests that were monitored through visits only. However, disturbance of breeding lapwings by nest monitoring procedures could have contributed to nest abandonment. Our 6 cm diameter poles were wider than the thin poles usually used to mark nests, and changes in the nest environment may have contributed to nest abandonment.

Nest abandonment could be associated with loss of an incubating parent, but we found no evidence for mortality among breeding adults. We suspect that abandonment was in at least one case related to the physiological stress of incubating in a hot climate. At one nest monitored late in the season at Alsace, mean daily soil temperatures exceeded 30°C for four consecutive days before peaking at 38.8°C. The nest was equipped with a camera, which recorded 37°C on the 16th of June 2022 at 14:00 with its internal thermometer. Footage showed an adult male panting in the shade of the maize plants directly next to the nest. The parent was observed incubating the clutch later the same evening, but the nest was abandoned the following day.

The abandoned nest illustrates some of the highest measured temperatures of our field seasons, but the means of daily peak soil temperatures of 27.4°C and 30.7°C for nest‐days in Hauts‐de‐France and Alsace were indicative of the generally warm conditions during the lapwing breeding season at our field sites. Eurasian Curlew (*Numenius arquata*) breeding at Lake Vyrnwy in Wales experienced mean maximum air temperatures of 12.8°C in April 2009 and 2010 and 14.2°C in May in the same years (Fisher and Walker 2015). For our lapwings' nest sites, the figures for air temperatures at a similar reference height are 17.8°C in April and 21.1°C in May. In Czechia, 20°C represented the 71% quantile of near‐surface temperatures during the lapwing breeding seasons of 2019 and 2020 (Koleškova et al. 2023), while at our field sites, 20°C represented the 71.4% quantile in Alsace and the 82.5% quantile in Hauts‐de‐France. For tundra‐breeding waders at high latitudes, surface temperatures may not reach above 28°C at all (Bulla et al. 2014).

Nests lost to agricultural operations related to crop management, such as tilling or planting, made up the greatest proportion of nest losses at our field sites (32% of confirmed nest failures), and the losses occurred significantly earlier in the breeding season compared to other causes of failure. Agricultural losses in Alsace (30% of nests) were similar to those for lapwings nesting on Norwegian crop fields between 1988 and 1990, where 43% of nests were lost to farming activities, with a majority of losses on untilled fields (Berg et al. 1992). Two potential explanations might explain the difference in nest losses to agriculture between our two regions. A difference in the start of the growing season between the two regions, or a difference in the detection of the seasons' first clutches. We do not have information on the timing of agricultural operations in our two regions, but a difference in timing is expected for the coastal climate in Hauts‐de‐France (Planchon 2000). A similar effect of climate on agricultural schedules has been previously noted at Polish crop fields, showing a 30‐day gradient in sowing dates at a national scale, with sites with a maritime climate initiating their growing season before more continental sites (Marcinkowski and Piniewski 2018). To further understand the timing of nest losses due to agricultural operations, it would be necessary to compare lapwings' breeding phenology and agricultural schedules for alternative crop types in the different regions.

A subset of early nests may have been missed in our field sampling. The earliest of our monitored nests hatched in mid‐April, but we also observed an adult with chicks in the first week of April 2021 on an island in a protected nature reserve, suggesting that nest initiation must have started in early March. In 2022, groups of migratory lapwings were already present in Alsace in mid‐February, with a few individuals spotted in the fields of Hauts‐de‐France. Early breeders could have started nests that were lost to agricultural operations in Hauts‐de‐France before they were discovered by field personnel.

The period of early April in Alsace was associated with high losses of nests to agricultural operations. Lapwing populations have declined from their peak numbers in this region of France (Dubois et al. 1991; Dronneau 2014), and future breeding success may depend on conservation efforts that focus on preventing reproductive losses at critical points in the breeding cycle.

### 4.2 Weather Impacts

Our best‐fit nest survival model predicted much lower nest survival rates at inland sites in Alsace than at coastal sites in Hauts‐de‐France. The regional difference was consistent with our prediction that Alsace would show lower rates of nest survival, given the historical population decline, and may be expected based on the regional differences in nest fates.

Lapwings breeding in France are near the southern boundary of the breeding range in Western Europe, and we expected that environmental conditions might be important drivers of reproductive success. Birds will abandon their nests at high temperatures, likely as a result of increasing thermoregulatory costs (Sharpe et al. 2019). In contrast to our predictions, we found that nest survival rates were not negatively impacted by dry weather or high temperatures. Instead, the best‐fit model indicated a positive relationship between daily mean wind speeds and daily nest survival. At higher wind speeds, convective heat loss of both birds and eggs increases (Bakken et al. 2002; Reid et al. 2002; Conover 2007). We might expect that the risk of abandonment will be reduced if greater heat loss is enabled through convection at higher wind speeds, and vice versa at lower wind speeds. At higher temperatures, birds will lose more water to thermoregulation (Eto et al. 2017). To prevent dehydration, lapwings would have to take incubation breaks to drink more often at high temperatures and low wind speeds, as nests at our study sites were often found some distance from surface water. Reduced nest attendance would result in a lower average number of lapwings in the breeding colony at any one time. Previous studies have shown that lower numbers of lapwings in a colony are associated with reduced anti‐predator responses (Elliot 1985).

The presence of an incubating shorebird attending a nest may serve as a cue for predators to locate its clutch (Engel et al. 2020), we might expect repeated movements between the nest site and a water source to serve as a similar cue. Additionally, thermoregulatory behaviour may increase the potential for visually oriented predators to detect the nest if lapwings assume a standing posture more often and for longer periods of time during times of high temperatures (Purdue 1976; Brown and Downs 2003). While standing, the parent's profile is raised, and concealment by surrounding habitat features is reduced (Hancock et al. 2023). The shadow of attending birds is also lengthened, the birds move, and the visually striking features of their plumage, including lapwings' white belly feathers and ruddy undertail covert feathers, are more conspicuous when held off the ground. The potential for olfactory predators to detect the nest may also be lower when wind speeds are higher, as higher wind speeds allow for odours to be dispersed more quickly (Conover 2007).

Lapwings breeding at high temperatures may thus experience a three‐fold benefit from higher wind speeds: (1) A reduced thermoregulatory cost through convective cooling, (2) a reduced risk of detection by predators due to less visual cues at the nest and faster dispersal of odours, and (3) a more effective anti‐predator response with a higher proportion of birds expected to be attending nests at the colony. A combination of these three effects may potentially explain why we found more support for a model using daily mean wind speeds, rather than daily temperatures.

We found that an interaction between wind speed and the wind speed of the previous day explained part of the nest survival rates of our nests. Survival rates were lower at the low end of daily wind speeds, but also when wind speeds were high for 2 days in a row. These combinations were likely related to inclement weather conditions. In combination with the effect of the monitoring method and region (Figure 4), it seems that differences between regions might affect the impact of the wind conditions at the nests. The accessibility of surface water features may provide alternative sources of cooling (Ryeland et al. 2021) and mitigate the need for a cooling breeze at the nest site. Field observations suggested that nests in Hauts‐de‐France were closer to water features than in Alsace. In addition, there may be differences in the cooling potential of the prevailing wind, which blows West to East from the English Channel in Hauts‐de‐France, but from SSW to NNE along the Rhine valley in Alsace (Davis et al. 2023). A sea breeze may be more effective at cooling than a land‐based south‐western wind. Future analyses might consider the interaction between wind speed and direction at nesting sites, or some measure of the expected cooling effect, as well as the availability of surface water for cooling and drinking.

If future research confirms our proposed mechanisms underlying the relationship between daily nest survival and weather conditions, we might expect that ground‐nesting birds will become more vulnerable to nest failure under a warming and drying climate (Gudmundsson and Seneviratne 2016; Guerreiro et al. 2018). Conservation authorities managing inland sites hosting breeding meadow birds might consider improving access to water. Access to water has known benefits for attracting breeding pairs to specific areas (Bertholdt et al. 2017; Van der Winden et al. 2017) and for providing resources for foraging chicks (Eglington et al. 2010), but could also provide a cooling resource during incubation that could improve the breeding success of Northern Lapwings under warming conditions in the future.

### 4.3 Monitoring Methods

We observed higher daily survival rates for nests equipped with cameras than for nests monitored with visits only. Additionally, there was an interaction effect between region and monitoring method, where the presence of cameras greatly increased nest survival in Alsace, but not in Hauts‐de‐France.

Previous studies have found varied effects of visiting nests of ground‐nesting birds, or marking the nest with a human object, on subsequent survival or depredation rates. Several species of prairie waders regularly nest beside conspicuous objects such as rocks, dirt mounds, or piles of manure, possibly to aid concealment of the incubating bird (Olson and Edge 1985; Coates et al. 2019). For some species and locations, artificial marking of nests does not appear to result in reduced nest survival (Galbraith 1987; Zámečník et al. 2018; Salewski and Schmidt 2022), whereas other studies have reported site‐specific negative or positive effects (Teunissen et al. 2008; Winder et al. 2016). Some studies have found no effect of the placement of a camera but a negative effect of visiting the nest (Stien and Ims 2016), while other studies have found no effect of frequent visitation when nests are left unmarked (Fletcher et al. 2005). Marking nests has been used to reduce losses to agricultural operations (Kragten and De Snoo 2007; Zámečník et al. 2018), but we were unsure whether to expect net‐positive or net‐negative effects on nest survival at our study sites if marking for nest protection was combined with an unpredictable effect on nest depredation rates.

Our net‐positive effect of camera placement on nest survival rates might be explained by local conditions at our study sites. First, there have been no nest marking programmes at our study sites using a setup similar to the one used in our study. Foxes and corvids often show some degree of neophobia (Miller et al. 2022; Morton et al. 2023), and the local novelty of our marking setup may have discouraged predators from approaching the nest sites. Second, a majority of our nests were found on crop fields, and most of those crop fields were planted with maize. Proposed mechanisms for altered depredation risk following human visits have included deposited scent trails or trails of disturbed vegetation (Teunissen et al. 2006). Maize fields at an early stage of growth can be accessed with minimal physical contact between researchers and crop plants, and were otherwise barren, so that local conditions may have limited trail deposition at the majority of our study sites. It is also possible that local predators generally avoid traces of human passage in our study areas, rather than investigating them. In this case, either monitoring method may have depressed depredation rates, although camera maintenance procedures likely caused more traces of human presence to be left at camera‐monitored nests.

To improve the reproductive output of lapwings at nesting sites in Alsace, significant nest marking effort may be required over multiple years, if not decades. For such marking programmes, nest outcomes might ideally be monitored to determine whether predators learn to associate a marking method with the presence of nests.

## 5 Conclusion

Our best‐fit model provided estimates of the probability for lapwing nests to survive incubation in Alsace that indicated considerable risks for nests in this region. The primary risk appears to be nest destruction through agricultural operations. To improve low rates of nest success in Alsace, local efforts to mark lapwing nests should be encouraged and supported. Marking and protection of lapwing nests has been supported by national agricultural programmes elsewhere in Europe (Zámečník et al. 2018), which may serve as a model to implement potential programmes in France. As the majority of nests lost to agriculture are lost in the early growing period, nest marking efforts in crop fields should be coordinated with local farmers and focus on the early part of the growing season. The key period would have been the first 2 weeks of April for the growing seasons in this study, but each year's “focus period” should depend on farmers' schedules in combination with that year's arrival dates of lapwings and weather conditions. Alternatively, use of predator‐deterring fences to exclude predators from the nesting and breeding areas may improve survival rates and reproductive rates when agricultural operations are managed appropriately (Verhoeven et al. 2022), though in some situations it may be best not to intervene at all (Goedhart et al. 2010). From our data, it appears that depredation is responsible for a low proportion of reproductive losses under current conditions at our field sites in France. As nest success was already high in Hauts‐de‐France, additional efforts to reduce nest losses may not be a productive investment of time and resources. Instead, where we have evidence of relatively high rates of nest survival, available resources may be better spent to investigate the subsequent survival of the chicks and resulting fledging rates. Continued observations would be useful to determine whether the breeding seasons of 2021 and 2022 in Hauts‐de‐France were particularly good for nest survival, or whether high rates of success are the usual condition. As more information on local demographic rates becomes available, a model‐based population viability analysis could provide insights into drivers of population dynamics and for identifying the best options for conservation. Population viability analyses have been successfully implemented for breeding lapwings in the Netherlands and Germany (Plard et al. 2020), and could serve as a basis for French efforts.

## Author Contributions

**Reinier F. Van den Berg:** conceptualization (equal), data curation (equal), formal analysis (equal), investigation (equal), methodology (equal), project administration (equal), resources (equal), visualization (equal), writing – original draft (equal), writing – review and editing (equal). **Brett K. Sandercock:** conceptualization (equal), formal analysis (equal), methodology (equal), writing – review and editing (equal). **Céline Le Bohec:** conceptualization (equal), funding acquisition (equal), project administration (equal), supervision (equal), writing – review and editing (equal). **Anna P. Nesterova:** conceptualization (equal), funding acquisition (lead), investigation (equal), methodology (equal), project administration (lead), resources (equal), supervision (lead), writing – review and editing (equal).

## Funding

This work was supported by Norges Forskningsråd (160022/F40) and Fédération Nationale Des Chasseurs.

## Conflicts of Interest

Funding for the field project was provided by the *Fédération Nationale des Chasseurs (FNC)*, the national association of French hunters. Lapwings are included on the quarry list of game species in France, and the FNC benefits from new information on the ecological conditions that are favourable for improving reproductive success and maintaining sustainable populations of lapwings in France. FNC received regular updates on project progress but was not involved with preparation or final approval of the project results.

## Data Availability

The data and code required for reproduction of the results are available on the figshare platform at https://doi.org/10.6084/m9.figshare.29920565.v2 with their accompanying descriptor file under the CC‐BY 4.0 licence (Van den Berg et al. 2025). Analysis code was verified to run successfully in an R environment (version 4.4.1) on 2025‐08‐19.
